# SARS-CoV-2 Infection and Placental Pathology

**DOI:** 10.1055/s-0041-1730291

**Published:** 2021-06-02

**Authors:** Caio Ribeiro Vieira Leal, Rayra Amana Macêdo Maciel, Mário Dias Corrêa Júnior

**Affiliations:** 1 Department of Gynecology and Obstetrics, Hospital das Clínicas, Faculdade de Medicina, Universidade Federal de Minas Gerais, Belo Horizonte, Minas Gerais, MG, Brazil

**Keywords:** SARS-CoV-2, Covid-19, pregnancy, placenta, pathology, SARS-CoV-2, Covid-19, gravidez, placenta, patologia

## Abstract

Placental pathophysiology in SARS-CoV-2 infection can help researchers understand more about the infection and its impact on the maternal/neonatal outcomes. This brief review provides an overview about some aspects of the placental pathology in SARS-CoV-2 infection. In total, 11 papers were included. The current literature suggests that there are no specific histopathological characteristics in the placenta related to SARS-CoV-2 infection, but placentas from infected women are more likely to show findings of maternal and/or fetal malperfusion. The most common findings in placentas from infected women were fibrin deposition and intense recruitment of inflammatory infiltrates. The transplacental transmission of this virus is unlikely to occur, probably due to low expression of the receptor for SARS-CoV-2 in placental cell types. Further studies are needed to improve our knowledge about the interaction between the virus and the mother-fetus dyad and the impact on maternal and neonatal/fetal outcomes.

## Resumo

A fisiopatologia da placenta na infecção por SARS-CoV-2 pode ajudar os pesquisadores a entender mais sobre a infecção e seu impacto nos resultados maternos/neonatais. Esta revisão breve fornece uma visão geral sobre alguns aspectos da patologia placentária na infecção por SARS-CoV-2. Ao todo, 11 artigos foram incluídos. A literatura atual sugere que não há características histopatológicas específicas nas placentas relacionadas à infecção por SARS-CoV-2, mas as placentas de mulheres infectadas têm maior probabilidade de apresentar achados de má perfusão materna e/ou fetal. Os achados mais comuns em placentas de mulheres infectadas foram deposição de fibrina e intenso recrutamento de infiltrado inflamatório. A transmissão transplacentária deste vírus é improvável, devido à baixa expressão do receptor para SARS-CoV-2 em tipos de células da placenta. Mais estudos são necessários para melhorar nosso conhecimento sobre a interação entre o vírus e a díade mãe-feto e o impacto nos resultados maternos e neonatais/fetais.

## Introduction

The most significant public health problem of the last decades is the coronavirus disease 2019 (Covid-19) pandemic, caused by the novel severe acute respiratory syndrome coronavirus 2 (SARS-CoV-2), responsible for more than 1 million deaths worldwide. 1 Some conditions have been associated with a higher risk of developing a severe illness, like advanced age, cardiovascular disease, diabetes mellitus, and hypertension. 2 However, data on the impact of the SARS-CoV-2 infection in pregnant women and in their fetuses or newborns are controversial. The available literature suggests that pregnant women have outcomes and clinical courses comparable to those of non-pregnant women of reproductive age, 3 4 and the newborns of infected mothers do not often show adverse clinical outcomes, 5 but there is few good-quality evidence to draw unbiased conclusions. 6 In any pregnancy infection, the placental pathophysiology can help researchers understand more about the disease and its impact on the maternal and neonatal outcomes. 7 The placenta is a transient pregnancy-related organ whose main function is to enable the maternal-fetal exchange of certain substances. 8 Some viruses can cross the placental barrier and infect the fetus, like the Zika virus, the cytomegalovirus, the rubella virus, and the herpesvirus. 9 So far, there is only one case report that showed unequivocal transplacental transmission of SARS-CoV-2. 10 There are many other papers about the vertical transmission of COVID-19, but no other convincing evidence has been found for the vertical transmission of this virus. 11 Besides being the possible key point for a fetal infection in pregnancy, the placenta itself can also be affected, morphologically and functionally, by the infection. 12 The aim of this brief review is to provide an overview about the data available in the literature about placental pathology in SARS-CoV-2 infection.

## Methods

This brief and non-systematic review was based on a search carried out independently by two authors (CRVL and RAMM) on the PubMed, Scopus, SciELO and Cochrane databases. The following search terms were used: *placenta* ; *placental pathology* ; *SARS-CoV-2* ; and *coronavirus* . Papers were selected after screening titles and articles. After data extraction and critical analysis, 11 case reports or series about placental alterations and pathophysiology in SARS-CoV-2 infection were included. 10 13 14 15 16 17 18 19 20 21 22

## Results

There are limited studies on SARS-CoV-2 infection and placental pathology. The most important aspects of each article found are shown in table 1 .

**Table 1:** Summary of papers about placental pathology in SARS-CoV-2 infection

Authors	Study characteristics	Main findings
Vivanti et al. 10	Case report of transplacental transmission of SARS-CoV-2 in a pregnant woman in the third trimester	The first case of proven transplacental transmission of SARS-CoV-2. The RT-PCR was positive for SARS-CoV-2 genes on the placenta, amniotic fluid and maternal, and fetal blood. Placental histological examination revealed diffuse perivillous fibrin deposition with infarction and acute and chronic intervillositis.
Hosier et al. 13	Case report of second trimester SARS-CoV-2-infected pregnancy complicated by severe preeclampsia and placental abruption	Placental histological examination showed diffuse perivillous fibrin deposition and an inflammatory infiltrate consistent with histiocytic intervillositis. There were no features of decidual vasculopathy. Placenta and umbilical cord tested positive for SARS-COV-2 RNA. Virus proteins were localized predominantly in the syncytiotrophoblast cells.
Hsu et al. 14	Case report of third trimester SARS-CoV-2-infected pregnant woman	Placental histological examination showed signs of maternal vascular malperfusion with hypertrophic arteriolopathy, but no fetal vascular malperfusion. There were signs of acute uterine hypoxia (subchorionic laminar necrosis) superimposed on chronic uterine hypoxia (extravillous trophoblasts and focal chronic villitis). Virus proteins were identified in chorionic villi endothelial cells and in trophoblasts.
Hecht et al. 15	Case series and comparative study between 19 SARS-CoV-2 infected pregnant women and 3 sets of controls	There was no specific gross or characteristic histopathology present in the placentas, including the only two infected placentas.
Smithgall et al. 16	Case series and comparative study between 51 SARS-CoV-2-infected pregnant women and 25 SARS-CoV-2-negative pregnant women	There were no specific histopathological characteristics in the placentas related to SARS-CoV-2 infection. None of the placentas tested positive for SARS-CoV-2. Maternal/fetal vascular malperfusion was identified in infected women, and their placentas showed more villous agglutination and subchorionic thrombi compared with non-infected women.
Facchetti et al. 17	Case series of 15 SARS-CoV-2-infected pregnant women	Only 1 of the 15 placentas tested positive for SARS-CoV-2 genes. The comparison between this placenta and the other 14 showed no significant morphological differences, except for the prominent intervillous inflammation (showing variable changes compatible with fetal vascular malperfusion).
Ferraiolo et al. 18	Case report of positive placental swabs for SARS-CoV-2 in an asymptomatic pregnant woman	Placental histological examination did not show substantial macroscopic alterations, except for mild to moderate subchorionic deposition of fibrin, for the presence of a single ischemic area in the thickness of the chorionic disc, for the appearance of villous agglutination, and for multiple organizing intervillous hemorrhages.
Shanes et al. 19	Case series and comparative study between 16 SARS-CoV-2 infected pregnant women and 2 populations of controls	Third-trimester placentas were significantly more likely to show decidual arteriopathy or at least one characteristic of maternal vascular malperfusion (MVM), such as abnormal or injured maternal vessels and intervillous thrombi, when compared to controls. Placentas were not tested for SARS-CoV-2.
Patanè et al. 20	Case series of 22 SARS-CoV-2-infected pregnant women in the third trimester	Only two newborns had SARS-CoV-2-positive nasopharyngeal swabs, whose placentas showed chronic intervillositis. On placental histological examination, no significant changes were observed in the other infected pregnant women.
Chen et al. 21	Case series of three SARS-CoV-2-infected pregnant women in the third trimester	Placental histological examination showed various degrees of fibrin deposition inside and around the villi, but no pathological change of villitis or chorioamnionitis. There were no specific placental morphologic changes related to SARS-CoV-2 infection.
Taglauer et al. 22	Case series and comparative study between 15 SARS-CoV-2-infected pregnant women and 10 SARS-CoV-2-negative pregnant women	There were no specific histopathological characteristics in the placentas related to SARS-CoV-2 infection. Placentas from infected women were notable for the presence of signs of inflammation and fibrin deposition, mostly intervillous and subchorionic deposition.

Abbreviations: RT-PCR, real-time polymerase chain reaction; SARS-CoV-2, severe acute respiratory syndrome coronavirus 2.

So far, there is no evidence that SARS-CoV-2 can induce specific histopathological changes in placentas. 15 16 21 22 Some studies identified SARS-CoV-2 proteins in placental tissues or cells. Facchetti et al. 17 identified the virus in the villous syncytiotrophoblast, endothelial cells, fibroblasts, in maternal macrophages, in Hofbauer cells, and in fetal intravascular mononuclear cells. Hosier et al. 13 and Patanè et al. 20 found the virus in the syncytiotrophoblast, and Hsu et al. 14 identified virus proteins in chorionic villi endothelial cells and in trophoblasts.

Vivanti et al. 10 presented the first proven case of transplacental transmission of SARS-CoV-2. A 23-year-old pregnant woman infected by SARS-CoV-2 was submitted to a cesarean-section in full isolation. Amniotic fluid was collected before membrane rupture and tested positive for SARS-CoV-2 genes, as well as the placenta and other maternal and fetal tissues.

The most common findings in the placenta of pregnant women infected with SARS-CoV-2 are fibrin deposition and intense recruitment of inflammatory infiltrates. Fibrin depositions have been observed in three different patterns: subchorionic deposition, 18 22 deposition inside the villi, 21 and perivillous deposition, 10 13 17 21 and the last pattern was the most observed. The intense inflammatory infiltrates were composed mainly of macrophages, 17 20 neutrophils, 17 T lymphocytes 13 14 and histiocytes. 14

In the study conducted by Smithgall et al., 16 51 third-trimester placentas from SARS-CoV-2-positive pregnant women (study group) and 25 third-trimester placentas from SARS-CoV-2-negative pregnant women (control group) were examined, and data were compared. As described before, no specific viral cytopathic modifications or evidence of vertical transmission were observed, but the study group showed evidence of maternal-fetal vascular malperfusion, with more villous agglutination ( *p* = 0.003) and subchorionic thrombi ( *p* = 0.026) than the control group. Ferraiolo et al. 18 also presented a case report of a third trimester SARS-CoV-2-positive placenta with villous agglutination.

Data also suggests that there is maternal and/or fetal malperfusion. Although the case reported by Hosier et al. 13 showed no decidual vasculopathy, the case report by Hsu et al. 14 demonstrated maternal vascular malperfusion (decidual hypertrophic arteriolopathy), with no fetal vascular malperfusion and, as aforementioned, Smithgall et al. 16 showed both maternal vascular malperfusion (decidual vasculopathy, intervillous thrombus, villus agglutination, and subchorionic thrombus) and fetal vascular malperfusion (avascular villi, fetal thrombotic vasculopathy, and chorangiosis). The study by Shanes et al. 19 indicated that placentas of SARS-CoV-2-positive pregnant women, compared to the control group (women with other medical conditions), were significantly more likely to exhibit intervillous thrombi ( *p* = 0.0002) and at least one feature of maternal vascular malperfusion ( *p* = 0,046), such as unusual or damaged maternal vessels.

The case series studied by Patanè et al. 20 presented 22 SARS-CoV-2-infected pregnant women. There were only two women whose newborns had SARS-CoV-2-positive nasopharyngeal swabs, and their placentas showed chronic intervillositis, accompanied by the existence of macrophages both in the intervillous and the villous spaces. Curiously, there were no significant alterations on the placenta of infected mothers whose newborns tested were negative.

## Discussion

To date, little is known about placental pathology in SARS-CoV-2 infection, but the current literature suggests that there are no specific changes in the placenta of infected pregnant women. As aforementioned, the most common findings in the placenta of SARS-CoV-2-infected pregnant women are fibrin deposition and intense recruitment of inflammatory infiltrates. Compared to controls, the placentas of infected women showed a higher probability of exhibiting intervillous thrombi and at least one feature of maternal vascular malperfusion, 19 more villous agglutination, and subchorionic thrombi. 16

Intervillous thrombi is the presence of a localized area of thrombosis in the chorionic villous stroma, while perivillous fibrinoid deposition is defined by the presence of fibrinoid material deposition in the intervillous space, and villous agglutination occurs when the distal villi are agglutinated by fibrin and bridging syncytial knots. 23 These patterns can be associated with processes of maternal malperfusion, such as placental insufficiency, fetal growth restriction, preeclampsia, thrombophilia, cardiovascular disease, renal abnormalities, or glucose intolerance. 23 24 25 26 Acute inflammatory lesions of the placenta are defined by diffuse infiltration of neutrophils and can involve every compartment of the placenta. 23 25 Chronic inflammatory lesions of the placenta are characterized by the infiltration of lymphocytes, plasma cells and macrophages, which may be a result of infections or may have an immune origin. 27 The main chronic inflammatory lesions of the placenta are villitis, chronic chorioamnionitis, and chronic deciduitis, 27 but chronic inflammation can also involve every compartment in the placenta, such as the intervillous space (intervillositis) or the umbilical cord (funisitis). 28 In our research, four articles showed two chronic inflammatory lesions in placentas from SARS-CoV-2-infected pregnant women: chronic intervillositis 10 13 20 and chronic villitis. 14 Both entities are usually reactions to infection, especially within the toxoplasmosis, other (syphilis, varicella-zoster, parvovirus B19), cytomegalovirus, and herpes simplex virus (TORCH) group, but when infectious causes are ruled out, they are called chronic intervillositis of unknown etiology (CIUE) and villitis of unknown etiology, both related to adverse obstetric outcomes, such as intrauterine growth restriction, preterm birth, and pregnancy loss. 28 29 30

Specifically when talking about viral infections, some patterns are well studied, such as the correlation between maternal cytomegalovirus infection to the presence of chronic lymphoplasmacytic villitis and hemosiderin deposition, 23 26 as well as some reports of nonspecific intervillositis in the setting of the Zika and Dengue virus. 19 It seems that there is no association between the presence of chronic or even acute specific inflammatory patterns and placental findings of SARS-CoV-2-infected women, 19 only nonspecific inflammatory infiltrates composed of macrophages, neutrophils, T lymphocytes and histiocytes, as aforementioned.

Data on placental pathology in diseases caused by other coronaviruses, the severe acute respiratory syndrome coronavirus (SARS-CoV) and the Middle East respiratory syndrome coronavirus (MERS-CoV), are scarce. Ng et al. 31 reported a case series of 7 placentas from pregnant women infected with SARS-CoV during the pandemic that occurred in Asia in 2003. Similar to SARS-CoV-2, there were no specific changes in those placentas. Two placentas of convalescent women who had the disease in the first trimester were normal, three placentas delivered in the acute stage of the disease showed increased subchorionic and intervillous fibrin deposition, and two placentas of convalescent women who had the disease in the third trimester showed extensive fetal thrombotic vasculopathy with sharply demarcated zones of avascular fibrotic villi (both had intrauterine growth restriction, oligohydramnios, and newborns small for gestational age). Data on MERS-CoV and placental pathology are even scarcer, but it seems that there is no relationship between this virus and specific placental disorders. 32

In regard to the vertical transmission of SARS-CoV-2, most studies 11 33 show that this mode of transmission is unlikely to occur. Only Vivanti et al. 10 could prove the transplacental transmission of this virus; therefore, if vertical transmission exists, it happens at low rates and possibly in selected cases. One of the cornerstones in this issue is how the virus infects the cells: through the angiotensin-converting enzyme 2 (ACE-2) receptor and the transmembrane serine protease 2 (TMPRSS-2), widely expressed in many tissues. 34 35 It is well established that the more the cell expresses ACE-2, the greater the chances it will be infected by coronaviruses. 36 There is no consensus about how much placental tissue express ACE-2 and TMPRSS-2. Taglauer et al. 22 showed a predominance of ACE-2 expression in comparison with TMPRSS-2 in placenta from infected women, but there was a significant decrease in ACE-2 expression in those placentas compared to those of non-infected pregnant women. Pique-Regi et al. 37 reported that placental tissues poorly express ACE-2 and TMPRSS-2, but receptors for other viruses that cause congenital infections (such as cytomegalovirus and the Zika virus) are highly expressed by placental cell types, and that is why vertical transmission for SARS-CoV-2 is unlikely to occur. The expression of ACE-2 in the placenta can be increased in some diseases, such as preeclampsia, 38 so there would be a theoretical increased risk of vertical transmission in this setting, for example. Additional studies are needed to evaluate the expression of ACE-2 and TMPRSS-2 in placental cells in physiological and pathological conditions to investigate the infection and transmission of SARS-CoV-2.

## Conclusion

In conclusion, in the present review, specific changes in the placentas of SARS-CoV-2-infected pregnant women were not found, but findings of maternal and/or fetal malperfusion were more likely to occur in infected than in non-infected women. The most common findings in the placentas from infected women were fibrin deposition and intense recruitment of inflammatory infiltrates. Little is known about placental pathology in SARS-CoV-2 infection, and further good evidence-based studies are needed in order to improve our knowledge about the interaction between the virus and the mother-fetus dyad and the impact on maternal and neonatal/fetal outcomes.
